# Frequency of c.35delG Mutation in* GJB2* Gene (Connexin 26) in Syrian Patients with Nonsyndromic Hearing Impairment

**DOI:** 10.1155/2017/5836525

**Published:** 2017-12-06

**Authors:** Hazem Kaheel, Andreas Breß, Mohamed A. Hassan, Aftab Ali Shah, Mutaz Amin, Yousuf H. Y. Bakhit, Marlies Kniper

**Affiliations:** ^1^Universitäts-HNO-Klinik Tübingen, Tübingen, Germany; ^2^Department of Bioinformatics, Africa City of Technology, Khartoum, Sudan; ^3^Faculty of Biotechnology, University of Malakand, Malakand, Pakistan; ^4^Department of Biochemistry, Faculty of Medicine, University of Khartoum, Khartoum, Sudan; ^5^Department of Basic Medical Sciences, Faculty of Dentistry, University of Khartoum, Khartoum, Sudan

## Abstract

**Background:**

Hearing impairments (HI) are the most common birth defect worldwide. Very large numbers of genes have been identified but the most profound is* GJB2*. The clinical interest regarding this gene is very pronounced due to its high carrier frequency (0.5–5.4%) across different ethnic groups. This study aimed to determine the prevalence of common* GJB2* mutations in Syrian patients with profound sensorineural HI.

**Methods:**

We carried out PCR, restriction enzyme based screening, and sequencing of 132 Syrian patients diagnosed clinically with hereditary deafness for different* GJB2* mutations.

**Results:**

The result revealed that, in* GJB2* gene, c.35delG is the most prevalent among affected studied subjects (13.64%), followed by c.457G>A (2.4%).

**Conclusion:**

The benefit of this study on the one hand is its first report of prelingual deafness causative gene mutations identified by sequencing technology in the Syrian families. It is obvious from the results that the deployment in biomedical research is highly effective and has a great impact on the ability to uncover the cause of genetic variation in different genetic diseases.

## 1. Introduction

Hearing impairments (HI) are one of the most common detected neonatal defects in the world [1]. Of every 1000 newborns, at least two have permanent bilateral sensorineural HI of more than 40 dB [2]. The causes of HI are equally shared between environment and genetics [3]. Genes causing HI are numerous and they cause isolated HI (nonsyndromic HI) in 70% of cases or in association with other medical abnormalities (syndromic HI) in the remaining 30% of cases [4]. 

HI occurs more in developing than developed countries [4]. Reported mutations occur more frequently in* GJB2* gene, which encodes the gap junction protein connexin 26 (beta-2,* GJB2*) [5].* GJB2* related HI is sometimes severe but the spectrum of variation severity is wide [6]. This gene is of special interest because of its high carrier frequency in many different ethnic groups (0.5–5.4%) [7, 8].

35delG is the most frequent mutation reported in the* GJB2* gene [8]. It is found in more than half of all* GJB2* mutations discovered so far [9]. This mutation creates a frameshift resulting in a short nonfunctional truncated form of the protein [10]. Additional mutations are continuously discovered worldwide but very few were done in Middle Eastern countries. This study aimed to determine the prevalence of common* GJB2* mutations in Syrians patients with profound sensorineural HI.

## 2. Material and Methods

This study was approved by the Ethical committee of the medical faculty (Syria). All subjects were recruited from Aleppo, which lies in the northwestern region of Syria, with the help of deafness related schools and institutes. The examined group consisted of 337 subjects (including 132 affected) from 54 families from Syria, where 2 or more subjects (up to 6) showed a prelingual, profound, sensorineural, bilateral, and nonsyndromic hearing loss within a family.

### 2.1. Study Population

In this study 175 females and 162 males were participated, with an age range between two and 72 years (mean age 18.2 years). A physical examination was performed to exclude syndromic forms of hearing impairment at Alrazi hospital (Aleppo), ENT examinations such as Tympanometry and BERA (Brainstem Electric Response Audiometry) were also applied at ENT department of the Alrazi hospital.

### 2.2. Mutation Screening

The mutation screening strategy was to investigate* GJB2* gene mutations including* 35delG* mutation (OMIM: 121011). First, total genomic DNA was extracted from 10 ml EDTA blood. This was done using a Qiagen Flexigen kits, in accordance with the manufacturer's instructions. By using primer pair [1F: 5′- AGA CTC AGA GAA GTC TCC CTG-3′/5R: 5′-GCC AGTTTA ACG CAT TGC CC-3′] the complete coding region of the exon of the GJB2 gene was amplified, in order to search for other possible mutations. The PCR product was gel extracted (Gel Extraction Kit, Qiagen) and sequenced using a capillary sequencer CEQ 8000 (Beckman Coulter) and the following PCR primers [2F: 5′-CCA GGC TGC AAG AAC GTG TGC-3′], [3R: 5′-GTG TAC CGG AGA CAT GAG-3′], [4F: 5′-GCA GCA TCT TCT TCC GGG TC -3′], [5R: 5′-GCC AGTTTA ACG CAT TGC CC-3′], 1F, and 5R. Detected mutations were confirmed at least two times and on both DNA strands compared to the reference sequence (Acc. No.: NM_ 004004).

*35delG Detection*. The frequency of 35delG variant was detected by PCR (35delG, F (forward): GGTGAGGTTGTGTAAGAGTTGG; 35delG, R (reverse): CTGGTGGAGTGTTTGTTCCCAC), followed by a BsiYI-digest [11]. Patients homozygous for 35delG showed one band with 181 bp and heterozygous patients displayed two bands (181 bp and 207 bp) after polyacrylamide gel electrophoresis (6%).

## 3. Results

Two different DNA sequence variants c.35delG and c.457G>A were detected in 54 Syrian families.

*c.35delG* variant was detected in 9 out of 54 (16.7%) families, 46 of 337 (13.64%) of subjects, of which 21 were homozygous and 25 were heterozygous for the variant (Table 1). 

*c.457G>A, (p.V153I) Variant*. We detected this variant in 2 out of 54 (3.7%) families, 8 of 337 (2.4%) subjects, who were only heterozygous for the variation.

## 4. Discussion

The purpose of this study was to identify the genetic basis of hereditary hearing impairment in Syrian patients with nonsyndromic HI, by applying Sanger sequencing leading to the results described above. The 35delG variant which is common worldwide was detected in 16.7% of Syrian families with nonsyndromic moderate to profound HI. Our results differ from a previous study by Al-Achkar et al. who found 35delG mutation in 30% of Syrian families, almost double that in our study [12]. This conflict is probably due to the difference in study areas. Geographical difference in prevalence of congenital deafness was also found previously in Italy [13].

35delG mutation takes place by deletion of one guanine (G) in a run of six guanines extending from position 30 to position 35 in the* GJB2* gene causing a frameshift of the coding sequence leading to premature chain termination at the twelfth amino acid [9]. Other studies in small groups found that this variant is responsible for nonsyndromic recessive deafness in a Muslim-Israeli village in the lower Galilee [14]. The frequency of 35delG variant is very high in Spain, Italy, and Israel [15]. It accounts for approximately 50% of cases [16] which suggests an evidence for an ancient deletion mutation that had spread in Europe and Middle-East.

The* c.457G>A *variant was found in about one-sixth of Syrian patients with HI. Same similar DNA variant had already been described in many other Asian countries [16, 17]. с.457G>A p.Val153Ile (rs11033186) in the ClinVar database is classified as a benign/likely benign variant. According to ExAc database world frequency of p.457G>A is 0.01057 (1279/121044 studied chromosomes, of which 922/16488 in South Asia and 299/66564 in Europe). However our results are not strong enough to change the annotation of this variant to likely pathogenic, and more studies are needed in this regard.

Based on these results we suggest a routine screening for* GJB2* gene mutations in Syrian population which could enhance the chance of detecting affected newborns with sporadic nonsyndromic HI at an early stage and consequently improves the current diagnostic and therapeutic options. Since* GJB2* gene has only one coding exon molecular diagnosis of mutations can be made simple and cost-effective for a daily medical practice. Important for future studies is an increase in selected sample sizes in order to obtain more information regarding possible novel genes and their mutations involved in the hearing process and their faults in hearing impairment.

## Figures and Tables

**Table 1:** Frequencies of 35delG genotypes among Syrian subjects with nonsyndromic hearing impairment.

Our selected group (familial cases)	c.35delG genotype	*N* (%)
Only the deaf	Homozygote WT^*∗*^	109 (82.6%)
	Heterozygote 35delG	2 (1.5%)
	Homozygote 35delG	21 (15.9%)
	Total	132
All subjects	Homozygote WT^*∗*^	291 (86.3%)
	Heterozygote 35delG	25 (7.4%)
	Homozygote 35delG	21 (6.2%)
	Total	337

^*∗*^WT: wild type.
